# Editorial: Host-pathogen interactions during pregnancy: Effects on maternal health, pregnancy outcomes, and fetal development

**DOI:** 10.3389/fcimb.2022.1010288

**Published:** 2022-09-08

**Authors:** Gregory W. Kirschen, Sylvie Girard, Karen Racicot, Irina Burd

**Affiliations:** ^1^ Department of Gynecology and Obstetrics, The Johns Hopkins Hospital, Baltimore, MD, United States; ^2^ Integrated Center for Fetal Medicine, The Johns Hopkins Hospital, Baltimore, MD, United States; ^3^ Department of Obstetrics and Gynecology, Mayo Clinic, Rochester, MN, United States; ^4^ Depatment of Gynecology & Obstetrics Quest Diagnostics, Secaucus, NJ, United States

**Keywords:** host, pathogen, pregnancy, Editorial, perinatal

This Research Topic edition includes several timely articles related to the interactions among maternal immune system and pregnancy physiology, host response to infection, and fetal development. One theme is using big data and advances molecular techniques to study the relationship between the maternal vaginal microbiome and pregnancy-related diseases.

Regarding the role of the vaginal microbiome in disease during pregnancy, Kumar et al. investigated the association between various vaginal bacteria, cytokine levels, and risk of Preterm Birth (PTB). Using 16S ribosomal RNA gene sequencing of vaginal cultures, they demonstrate that patients who experienced PTB exhibited higher levels of *Prevotella buccalis*, and lower levels of *Lactobacillus crispatus and Finegoldia*. They also observed alterations in cytokine levels in these patients, including depressed levels of IFNγ, IL-4, and TNFα. They conclude that alterations in vaginal microbial diversity and local vaginal immune suppression may contribute to PTB.

Aside from its role in response to pathogen invasion, the immune system is responsible for normal physiological processes that allow for the onset and progression of labor. Leimert et al. review how sterile inflammation is required for dynamic uterine and cervical changes that allow for cervical dilation, uterine contraction, and ultimately birth (or preterm birth). Sterile inflammation is also implicated in the pathogenesis of preeclampsia, a hypertensive disorder of pregnancy. Banerjee et al. review the evidence that preeclampsia may stem from an abnormal immune response without infection. Damage-associated molecular patterns (DAMPs) and Alarmins released by the placenta in preeclampsia prompt endoplasmic reticulum (ER) stress, dysregulated autophagy, and inflammatory cascades resulting in multiorgan dysfunction. Chemokines and uric acid build up, affecting vascular smooth muscle function, contributing to systemic vasoconstriction and hypertension characteristic of preeclampsia. Women with preeclampsia are also at risk for spontaneous preterm birth, suggesting that aberrant sterile inflammation may underlie both processes (Haedersdal et al., 2013).

While sterile inflammation plays a role in labor and preeclampsia, infection-associated inflammation also commonly occurs in pregnancy and labor. Chorioamnionitis refers to an ascending infection from the lower female urogenital tract in pregnancy affecting the placenta, amniotic membrane, or both. Chorioamnionitis is characterized by a polymicrobial infection, formation of a biofilm, and maternal and fetal host responses to the invading pathogens (Lutz et al.) Chorioamnionitis is also an important contributor to preterm labor and PTB, and adds significantly to the associated perinatal morbidity and mortality (Tita and Andrews, 2010). Lutz et al. review various animal models of human chorioamnionitis, including rodent, sheep, and non-human primate models, detailing how each has contributed to our understanding of the pathogenesis of this disease. The authors explain various steps involved in producing the infection and host response, starting with ascending infection, leading to primary intrauterine inflammation, polymicrobial infection, fetal inflammatory response, and resulting PTB. One infectious agent that can lead to chorioamnionitis and associated neonatal sepsis is group B streptococcus (GBS). Brokaw et al. review the shifts in maternal vaginal microbiome that occur in cases of GBS colonization. On the other hand, certain bacterial flora may be protective against GBS colonization and infection, including Gardnerella vaginalis, Prevotella, Dialister, and Megasphaera species.

Bacteria are not the only pathogens that can cause changes in maternal immune responses and inflammation/infection. Viruses are also an important cause of congenital infection. Hutton and Rowen highlight in their original research article how vertical transmission of cytomegalovirus (CMV), an important infectious cause of congenital hearing loss and neurodevelopmental delay, can be influenced by number of gestations. They find that the rate of congenital CMV transmission from mother to fetus is elevated in twin versus singleton gestations. Heritability of vertical transmission is high, suggesting that genetic factors may play an important role in risk of maternal to fetal transmission of the virus.

Another virus that has been the subject of much research and investigation over the past two years is SARS-CoV2. This virus is the causative pathogen of COVID-19 infection, which affects pregnant individuals just as it does non-pregnant individuals. Chen et al. review major immune changes that occur in the pregnant state and these changes can lead to severe consequences in the setting of SARS-CoV2 infection in pregnancy. Innate immunity is blunted in pregnancy, likely due to the anti-inflammatory effects of estradiol, which is heightened in pregnancy and inhibits production of IL-6 and IL-1β, key cytokines in the host response to SARS-CoV2. With pathogen exposure, lower levels of pro-inflammatory cytokines are released and there is less robust phagocytic activity, as compared to the non-pregnant state, compromising the host response to SARS-CoV2-infected cells. In particular, the heightened respiratory morbidity and associated mortality in pregnant women as compared to non-pregnant women with COVID-19 infection can be partially attributed to depressed NK cell populations in pregnancy, with these cells playing an important role in defending against pulmonary infection (Troiano et al., 2022). Furthermore, not only do absolute T helper and T killer cell counts decrease in pregnancy, but their activity when faced with viral pathogens such as SARS-CoV2 also decreases (Chen et al.).

With regard to COVID-19, there has been a great emphasis on whether vaccination is safe and effective for pregnant and breastfeeding women. Shook et al. review the current state of the evidence surrounding our understanding of COVID-19 vaccination in pregnant and breastfeeding women. They firstly describe that safety and reactogenicity profiles are not significantly different between these women and non-pregnant, nonlactating women, based on observational studies and vaccine safety monitoring programs. They also describe that the serological response to the COVID-19 mRNA vaccine in the pregnant state is comparable to that of the non-pregnant state. These results are encouraging, and are reflected in the official recommendations of the American College of Obstetricians and Gynecologists (ACOG) and the Society for Maternal Fetal Medicine (SMFM), which both encourage vaccination in pregnant women (ACOG, 2021).

In summary, this edition of the journal highlights our improved understanding of fundamentals of pregnancy physiology, from how the onset of labor is triggered by sterile inflammation, to how the maternal vaginal microbiome and immune system interact with pathogens resulting in colonization, infection, vertical transmission, and potentially PTB and neonatal consequences.

## Author contributions

GK and IB wrote the original draft. All authors proofread and edited the final draft and agree with the final version.

## Funding

This work was funded through the National Institutes of Health (NIH) award 5R01HD097608-04 to IB and through internal departmental funding (Kelly Society Award) to GK.

## Conflict of interest

The authors declare that the research was conducted in the absence of any commercial or financial relationships that could be construed as a potential conflict of interest.

## Publisher’s note

All claims expressed in this article are solely those of the authors and do not necessarily represent those of their affiliated organizations, or those of the publisher, the editors and the reviewers. Any product that may be evaluated in this article, or claim that may be made by its manufacturer, is not guaranteed or endorsed by the publisher.
